# Porphyrin *N*-Pincer Pd(II)-Complexes in Water: A Base-Free and Nature-Inspired Protocol for the Oxidative Self-Coupling of Potassium Aryltrifluoroborates in Open-Air

**DOI:** 10.3390/molecules26175390

**Published:** 2021-09-04

**Authors:** Sana Siva Prasad, Bandameeda Ramesh Naidu, Marlia M. Hanafiah, Jangam Lakshmidevi, Ravi Kumar Marella, Sivarama Krishna Lakkaboyana, Katta Venkateswarlu

**Affiliations:** 1 Laboratory for Synthetic & Natural Products Chemistry, Department of Chemistry, Yogi Vemana University, Kadapa 516005, India; sana.chemist009@gmail.com (S.S.P.); brameshnaidu94@gmail.com (B.R.N.); devi703244@gmail.com (J.L.); 2 Department of Earth Sciences and Environment, Faculty of Science and Technology, Universiti Kebangsaan Malaysia, Bangi 43600, Selangor, Malaysia; mhmarlia@ukm.edu.my; 3 Centre for Tropical Climate Change System, Institute of Climate Change, Universiti Kebangsaan Malaysia, Bangi 43600, Selangor, Malaysia; 4 Department of Chemistry, PACE Institute of Technology & Sciences, Ongole 523272, India; ravikumar.marella@pace.ac.in; 5 Department of Chemical Technology, Chulalongkorn University, Pathumwan, Bangkok 10330, Thailand; svurams@gmail.com

**Keywords:** porphyrin *N*-pincer Pd(II)-complexes, water, nature-inspired conditions, potassium aryltrifluoroborates, self-coupling, symmetrical biaryls

## Abstract

Metalloporphyrins (and porphyrins) are well known as pigments of life in nature, since representatives of this group include chlorophylls (Mg-porphyrins) and heme (Fe-porphyrins). Hence, the construction of chemistry based on these substances can be based on the imitation of biological systems. Inspired by nature, in this article we present the preparation of five different porphyrin, *meso*-tetraphenylporphyrin (**TPP**), *meso*-tetra(*p*-anisyl)porphyrin (**T*p*AP**), tetrasodium *meso*-tetra(*p*-sulfonatophenyl)porphyrin (**TST*p*SPP**), *meso*-tetra(*m*-hydroxyphenyl)porphyrin (**T*m*HPP**), and *meso*-tetra(*m*-carboxyphenyl)porphyrin (**T*m*CPP**) as well as their *N*-pincer Pd(II)-complexes such as Pd(II)-*meso*-tetraphenylporphyrin (**PdTPP**), Pd(II)-*meso*-tetra(*p*-anisyl)porphyrin (**PdT*p*AP**), Pd(II)-tetrasodium *meso*-tetra(*p*-sulfonatophenyl)porphyrin (**PdTST*p*SPP**), Pd(II)-*meso*-tetra(*m*-hydroxyphenyl)porphyrin (**PdT*m*HPP**), and Pd(II)-*meso*-tetra(*m*-carboxyphenyl)porphyrin (**PdT*m*CPP**). These porphyrin *N*-pincer Pd(II)-complexes were studied and found to be effective in the base-free self-coupling reactions of potassium aryltrifluoroborates (PATFBs) in water at ambient conditions. The catalysts and the products (symmetrical biaryls) were characterized using their spectral data. The high yields of the biaryls, the bio-mimicking conditions, good substrate feasibility, evading the use of base, easy preparation and handling of catalysts, and the application of aqueous media, all make this protocol very attractive from a sustainability and cost-effective standpoint.

## 1. Introduction

**Scheme 1:**
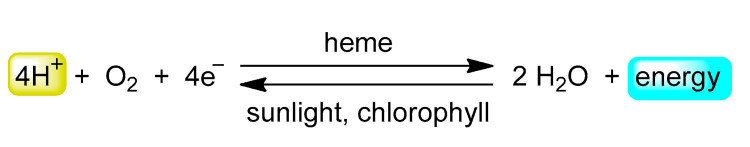
Schematic representation of metalloporphyrin catalyzed basic reactions of life.

**Figure 1:**
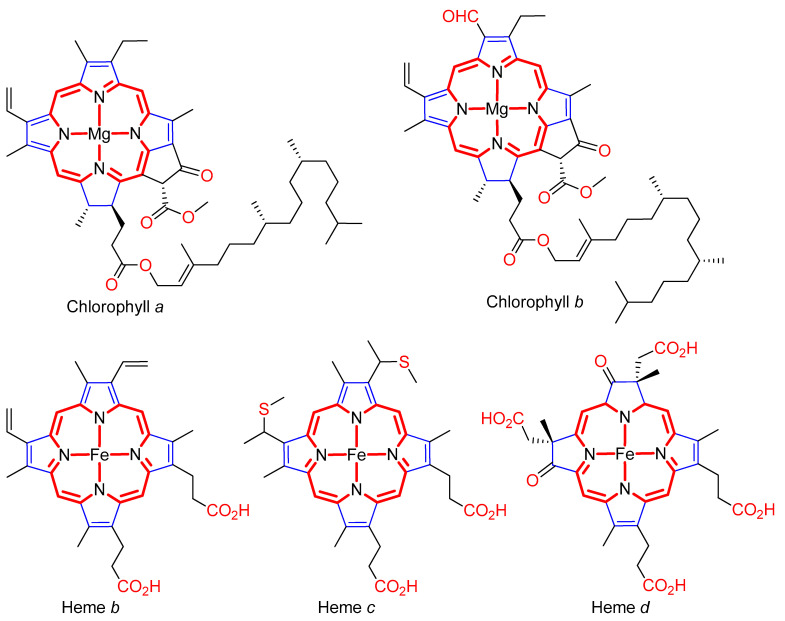
Structures of some metalloporphyrins which demonstrates the life on earth.

Synthetic and natural metalloporphyrins are well-known examples of nitrogen-bridged polycyclic compounds and are largely distributed in nature. Metalloporphyrins display a critical role in numerous biological tasks including oxygen transport, health sustainment, bio-organic transformations, and light-harvesting [1,2,3]. Metalloporphyrins display vital catalytic significance in the basic reactions of life; for example, as heme (a cofactor of hemoglobin) in oxygen transport [1,4] and as chlorophyll (which is able to convert sunlight to energy) in photosynthesis of plants [4,5] (Scheme 1). Several examples of heme and chlorophylls are displayed in Figure 1. Despite these efficient biochemical, photochemical, and enzymatic functions, the connectivity and controlled rigidity of metalloporphyrins allow highly ordered arrangements in their crystalline frameworks; for example, metal-organic frameworks (MOFs) encompassing an exciting area of research for over a decade in chemical science and technology disciplines [1,5,6].

Besides the biological-catalytic functions of metalloporphyrins, their aptness to a large number of organic transformations as effective catalysts is well-documented [2,7] and is due to the wide selection of metal-ions that can form complexes with porphyrins [2]. The metalloporphyrins have been reported for their catalytic efficiency in cross-coupling reactions [2,8,9,10,11,12], epoxidations of alkenes [7,13,14], oxidation of alcohols/thiols/benzylic groups/aldehydes [15,16,17], cycloaddition reactions [18,19], reductions of multiple bonds [20,21], aziridinations of olefins [22,23], and olefin cyclopropanations [24,25]. These metalloporphyrins have also been reported as effective catalysts in large scale organic transformations [7] which is an additional benefit together with their inherent safeness and biomimicking properties [2,12]. Hence, the application of metalloporphyrins as catalysts in further organic reactions is interesting and imitates organic reactions of nature.

Self-coupling is an important strategy among the existing procedures for symmetrical biaryl synthesis and is a straightforward and convenient process [26]. This method avoids the requirement of two different substrates that are normally required in cross-coupling procedures [27]. Aryldiazonium salts [26,28,29], aryl halides [26,30,31,32], arylboronic acids [26,27,33,34,35], arylboronates [26,35,36,37,38,39,40,41,42,43], arylmagnesium compounds [26,44,45], aryllithium compounds [26,46,47], arylmercury salts [26,48,49], aryl mesylates [26,50,51,52], aryl tosylates [26,52], aryl triflates [26,52,53], arylsilanes [26,54,55], arylcarboxylic acids [26,56,57], and tetraarylborates [58,59,60,61] are the substrates used for this self-coupling process. In this connection, aryl halides, arylcarboxylic acids, arylboronic acids, arylsilanes, and arylboronates seem to be stable substrates [26,27]. Among these self-couplings, the couplings of arylcarboxylic acids, arylsilanes, and aryl halides require harsh conditions like oxidants, high temperature, and co-catalysts [26,27], but the self-coupling of arylboron substrates can be performed at room temperature (rt) and (or) using mild conditions [26,27,33,34,35,36,37,38,39,40,41,42,43]. The application of aryltrifluoroborates in self-coupling reactions is underdeveloped despite the high stability, fair water solubility, and good reactivity of these substrates [10,39,40,41,42,43]. 

The catalysts based on transition-metals such as Pd [26,27,33,34,35,36,37,38,39,41,43], Au [40,62,63,64], Cu [42,65,66,67,68], Rh [69], Ru [70], and Fe [71] have been reported for the self-coupling transformations of arylboron compounds but most of the Au, Cu, Rh, Ru, and Fe-based reactions suffer from drawbacks such as the requirement of an external oxidant, a base, organic solvent, low product yields, formation of by-products, and high temperature [26,27]. The Pd-promoted methods, on the other hand, can be performed at rt using mild reaction conditions in a safer solvent such as water [27]. Hence, we undertook the development of a new Pd-based protocol for the synthesis of biaryl using a self-coupling strategy, and found *N*-pincer Pd(II)-porphyrin complexes as efficient catalysts for this purpose in water, employing PATFBs as attractive substrates. The present protocol using water as reaction media (which is nature′s preferred solvent instead of flammable, volatile, and toxic organic solvents [72]), together with metalloporphyrins as catalysts (which are the catalysts of several significant functions in biology), at ambient conditions in open-air can become a nature mimicking protocol for the synthesis of symmetrical biaryls.

## 2. Results and Discussion

**Figure 2:**
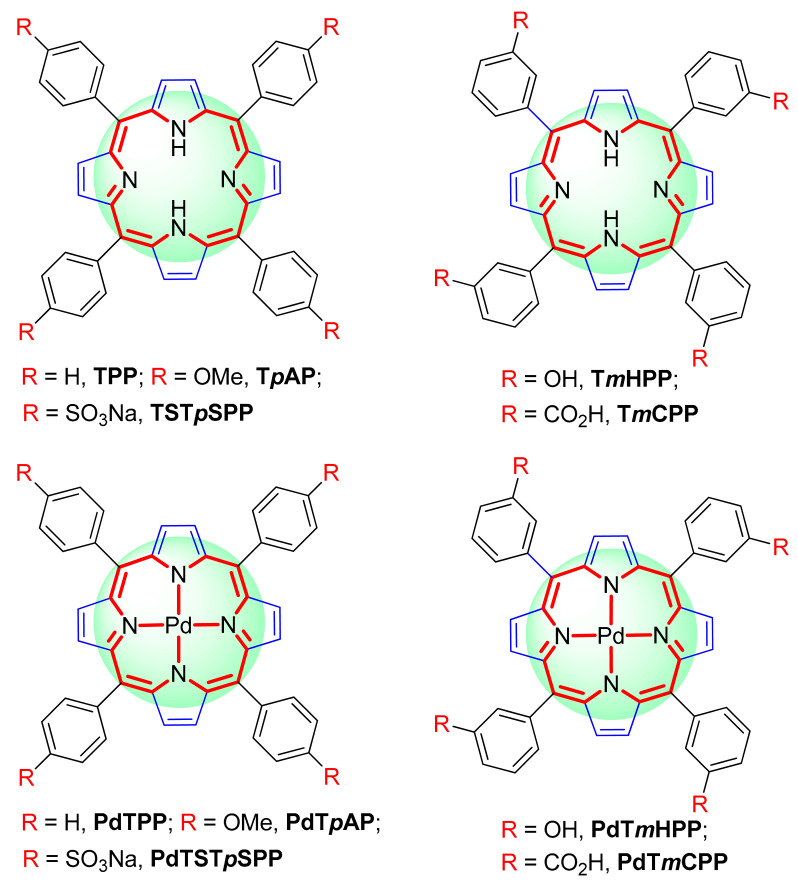
Chemical structures of porphyrins and their *N*-pincer Pd(II)-complexes (catalysts).

The porphyrins, **TPP**, **T*p*AP**, **TST*p*SPP**, **T*m*HPP,** and **T*m*CPP**, and their *N*-pincer Pd(II)-complexes, **PdTPP**, **PdT*p*AP**, **PdTST*p*SPP**, **PdT*m*HPP,** and **PdT*m*CPP** (Figure 2) were synthesized according to our previous reports [2,10,11,12] (Section 3.1.2 and Section 3.1.3). The characterization data of these compounds were revealed in our published data [10].

Initially, potassium 4-methoxyphenyltrifluoroborate (**1a**) was found to participate in self-coupling to give 98% of biaryl, **2a** (in 15 min) using 0.1 mol% of **PdTST*p*SPP**, 4 mL of water at rt in open-air (entry 1, Table 1). This transformation using **PdTPP**, **PdT*p*AP**, **PdT*m*HPP,** and **PdT*m*CPP**, each with 0.1 mol% in 4 mL 1:1, vol:vol mixture of water and DMF was observed to provide **2a** in 21%, 39%, 58%, and 67% in 4h and indicated that the catalyst, **PdTST*p*SPP** was highly suitable for the self-coupling of **1a** (entries 2–5, Table 1). Further, the catalyst loading studies, using 0.07 mol%, 0.05 mol%, and 0.03 mol% showed the formation of **2a** in 98%, 98%, and 82% in 15 min, 15 min and 60 min (entries 6–8, Table 1), suggesting the requirement of 0.05 mol% **PdTST*p*SPP** as catalyst for the self-coupling of **1a**. The investigations using other arylboron compounds such as, 4-methoxyphenyboronic acid (**2**), neopentylglycol, and pinacol esters of 4-methoxyphenylboronic acid (**3** and **4**) and the diethanolamine derivative of 4-methoxyphenylboronic acid (**5**) showed 92%, 14%, 17%, and 73% yields of **2a** (entries 9–12, Table 1), indicating that potassium 4-methoxyphenyltrifluoroborate (**1a**) is the best substrate for the current self-coupling process. This may be due to the high water solubility of the aryltrifluoroborates.

The applicability of this nature-inspired procedure has also been studied using a variety of aryltrifluoroborates (**1a**–**1u**). The PATFBs with electron-releasing functionalities (ERFs) such as -OMe, -Me, -Br, -OH, -SMe, and -*^t^*Bu at *p*-, *m*- and *o*-positions delivered excellent yields (88–98%) of the self-coupling products, **6a**–**6f**, **6l**, **6m** and **6p** with high turnover number (TON) (1760–1960) and turnover frequency (TOF) (2347–7840) values (entries 1–6,12,13,16, Table 2). Electron withdrawing functionalities (EWFs) containing PATFBs at all the *p*-, *m*-, and *o*-positions were found as the best substrates to give 91–99% of self-coupled products, **6g**–**6k**, **6n**, **6o,** and **6q** with large values of TON as 1820–1980 and TOF as 3680–11880 (entries 7–11,14,15,17, Table 2). Unsubstituted PATFB such as **1r** and potassium salts of heteroaryltrifluoroborates, **1s**–**1u** also provided excellent isolated yields of self-coupled products under *N*-pincer Pd(II)-porphyrin, **PdTST*p*SPP** catalyzed reactions in water with excellent yields (86–96%) of products, **6r** and **6s**–**6u** with TON, 1720–1920 and TOF, 1720–7680 (entries 18–21, Table 2). This study revealed that the current **PdTST*p*SPP** catalyzed self-coupling of PATFBs shows a large substrate scope irrespective of position and nature of the functional groups. The structures of all the symmetrical biaryls were confirmed using their ^1^H NMR, ^13^C NMR and mass (LCMS) spectral data (Section 3.2) and the copies of the ^1^H NMR and ^13^C NMR spectra has been provided at Supplementary Materials with this article.

**Scheme 2:**
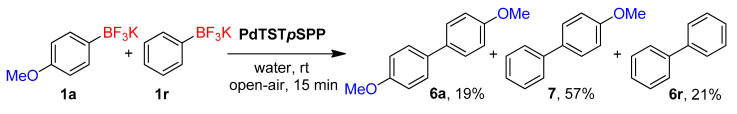
Study of **PdTST*p*SPP** catalyzed hetero-coupling of PATFBs.

We also studied the hetero-coupling reaction of PATFBs, **1a** and **1r** (each with 0.5 mmol) under the present conditions, and observed the formation of self-coupling products **6a** and **6r** along with the hetero-coupling product **7**, in 19%, 21%, and 57% yields in 15 min (Scheme 2). This study indicated that the developed method shows some selectivity in the formation of hetero-coupling products over self-couplings, and hence a detailed investigation may be undertaken towards a complete understanding of the hetero-couplings of arylboron compounds using metalloporphyrin-based catalysts.

**Scheme 3:**
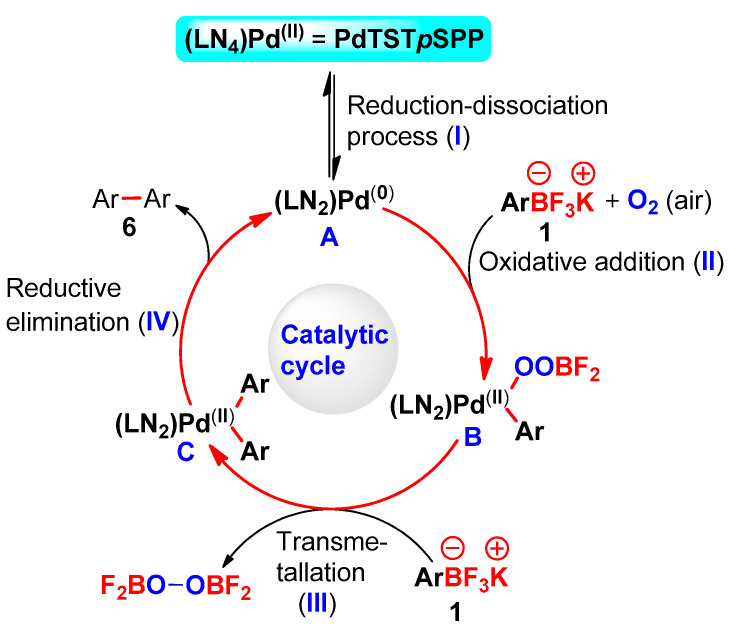
Proposed mechanism of **PdTST*p*SPP** catalyzed self-coupling of PATFBs.

The plausible mechanistic futures of **PdTST*p*SPP** catalyzed self-coupling of PATFBs is sketched in Scheme 3 based on previous reports [10,11,12,27,39,73]. The reduction-dissociation process of **PdTST*p*SPP** delivers the Pd(0)-porphyrin intermediate **A [10,11,12]**. The Pd(0)-porphyrin species **A** is involved in oxidative addition with PATFB **1** and atmospheric oxygen to give Pd(II)-species **B**, which is on transmetallation with **1** gives the diarylPd(II) intermediate **C** [27,39,73]. Intermediate **C** forms symmetrical biaryl **6** and Pd(0)-porphyrin active catalytic principal **A** on reductive elimination.

A comparison of reported self-coupling procedures of aryltrifluoroborates [39,40,41,42,43] is shown in Table 3 and evidences the clear merits of the present nature-mimicking method over the reported protocols using Pd NPs/Te-Dps [39], Au nanoclusters:poly(*N*-vinyl-2-pyrrolidine) [40], Pd(OAc)_2_–electrolysis [41], Cu(OAc)_2_–ultra sound [42], and Pd NPs@Al(OH)_3_ [43] which require organic solvent [41], base/additive [39,40,41,42,43], heating [39,40,41,42,43], long process time [39,40,42,43] or suffer from low biaryl yield with some aryltrifluoroborates [39,40,41,42,43]. Hence, the present *N*-pincer Pd(II)-porphyrin catalyzed process is advantageous over the reported aryltrifluoroborate self-couplings. In view of global sustainability, the elimination/decrease of the application of volatile organics, the use of benign/nature-mimicking catalysts, and conducting the chemical reactions at ambient conditions can make a significant contribution [74,75,76,77,78]. 

## 3. Materials, Methods and Characterization Data

### 3.1. Materials and Methods

#### 3.1.1. General

The chemical substances utilized in the present homocoupling of PABs were purchased from Spectrochem (Mumbai, India), Alfa Aesar (Haverhill, MA, USA), Merck (Burlington, MA, USA), AVRA (Hyderabad, India), Sigma-Aldrich (St. Louis, MO, USA), and TCI (Tokyo, Japan). Porphyrins and Pd(II)-porphyrin complexes were made from literature reports [2,10,79]. Pyrrole was directly purified by distillation before its use. Silica gel coated thin layer chromatography (TLC) (Merck, Burlington, MA, USA, silica gel-60 F_254_) was employed to confirm the progress of the self-couplings. Silica gel-packed glass-columns were employed to produce the pure symmetrical biaryls using an eluent of a mixture of EtOAc and hexanes. The Bruker Avance 400/100 MHz NMR spectrometer (Billerica, MA, USA) was employed to record the ^1^H and ^13^C-NMR spectra and molecular mass was recorded with a Thermo LCQ Max LCMS (Dreieich, Germany).

#### 3.1.2. Synthesis of Porphyrins 

**TPP**: Propanoic acid (180 mL) at 140 °C was added to 75 mL of pyrrole and 8.37 g of benzaldehyde, and the mixture was heated for 1h at 140 °C. The reaction contents were cooled to rt then 110 mL of EtOH was added and stirred at rt for 1 h. The reaction mixture was filtered and the filtrate evaporated in vacuo. Finally, the residue obtained was used to purify the TPP using neutral alumina-packed-column chromatography (CC) with eluent CHCl_3_.

**TpAP**, **T*m*HPP,** and **T*m*CPP**: Propionic acid (180 mL) was added to 7.30 g of its anhydride and heated for 5 min at 140 °C. Then, 5.00 g of pyrrole (distilled), and 80 mmol of *p*-anisaldehyde/*m*-hydroxybenzaldehyde/*m*-formylbenzoic acid were added, stirred at 140 °C for 1 h and the mixture cooled to rt. Then 100 mL of EtOH was added, stirred at rt for 1 h, and filtered. The obtained residue was dried in vacuo and subjected to neutral alumina-packed-CC with eluent CHCl_3_ to obtain the pure porphyrins, **T*p*AP**, **T*m*HPP**, and **T*m*CPP**.

**TST*p*SPP**: TPP (5 gr) in conc. H_2_SO_4_ (60 mL) was heated at 60 °C 16 h and cooled to rt with 12 mL of added ice-cold water. The obtained green colored solution was adjusted to pH between 9–10 using an aqueous solution of saturated NaHCO_3_. The mixture was evaporated in vacuo and thoroughly washed using 2 × 25 mL of CH_2_Cl_2_. A solid precipitate obtained on the addition of 20 mL of MeOH:acetone (3:7) was separated, dried in vacuo, and the **TST*p*SPP** was subjected to purification using neutral Al_2_O_3_-packed-CC with the eluent, MeOH:acetone (3:7).

#### 3.1.3. Synthesis of *N*-pincer Pd(II)-porphyrin Complexes 

**PdTPP**, **PdT*p*AP**, **PdT*m*CPP,** and **PdT*m*CPP** [10]: 5 mmol of porphyrin (**TPP**/**TpAP**/**TmHPP**/**TmCPP**), 7.5 mmol of PdCl_2_ in 20 mL DMF were refluxed for 2 h, cooled to rt, and filtered. The filtrate was diluted with 40 mL EtOAc, washed using 2 × 20 mL of water and 2 × 15 mL of brine. EtOAc was evaporated in vacuo and the resultant residue of the Pd-porphyrin complexes subjected to purification using CC with eluent, MeOH:CH_2_Cl_2_ (5:95).

**PdTSTpSPP [10]**: **TST*p*SPP** (2.56 g, 2.5 mmol), PdCl_2_ (0.53 g, 3 mmol) in 12 mL DMF was refluxed for 2 h and cooled to rt and subjected to evaporation to obtain a dried reaction mass. The crude solid was employed for the purification of **PdTSTpSPP** using CC with the eluent acetone:MeOH (8:2).

#### 3.1.4. PABs Homocoupling Procedure

To **PdTST*p*SPP** (0.05 mol%) and PATFB (**1**) (1.10 mmol), 4 mL of deionized water was added and the mixture stirred at rt in open-air for the appropriate time (Table 2). To ensure the completion of the reaction by TLC, to the reaction mixture was added 5 mL water and it was extracted using EtOAc (2 × 5 mL). The EtOAc combined solution was dried in vacuo and subjected to silica-gel packed-CC to obtain the pure symmetrical biaryls (**6**). The products (**6**) structures were determined by their ^1^H and ^13^C NMR and mass data. The characterization data of **6** (Section 3.2) was found to be similar to that of that reported [27,30,66,67] and the copies of ^1^H and ^13^C NMR spectra has been provided as Supplementary Materials with the manuscript.

### 3.2. Characterization Data of Symmetrical Biaryls

**6a [27,30]**: ^1^H NMR (400 MHz, chloroform-*d*_6_): δ (ppm) = 7.46 (d, *J* = 7.4 Hz, 4H, Ar-H), 6.94 (d, *J* = 7.4 Hz, 4H, Ar-H), 3.83 (s, 6H, -OMe); ^13^C NMR (100 MHz, chloroform-*d*_6_) δ (ppm) = 158.8, 133.6, 127.8, 114.2, 55.4; LCMS (*m*/*z*): 215 (M + H); Formula: C_14_H_14_O_2_.

**6e [27]**: ^1^H NMR (400 MHz, chloroform-*d*_6_): δ (ppm) = 7.49 (d, *J* = 7.3 Hz, 4H, Ar-H), 7.30 (d, *J* = 7.3 Hz, 4H, Ar-H), 2.51 (s, 6H, -SMe); ^13^C NMR (100 MHz, chloroform-d_6_) δ(ppm) = 137.6, 137.4, 127.2, 127.1, 16.0; LCMS (*m*/*z*): 247 (M + H); Formula: C_14_H_14_S_2_.

**6f [27]**: ^1^H NMR (400 MHz, chloroform-d_6_): δ (ppm) = 7.54–7.49 (m, 4H, Ar-H), 7.46–7.42 (m, 4H, Ar-H), 1.35 (s, 18H, -*^t^*Bu); ^13^C NMR (100 MHz, chloroform-d_6_) δ(ppm) = 150.0, 138.3, 126.7, 125.7, 34.6, 31.5; LCMS (*m*/*z*): 267 (M + H); Formula: C_20_H_26_.

**6h [27,67]**: ^1^H NMR (400 MHz, chloroform-*d*_6_): δ (ppm) = 7.49–7.46 (m, 4H, Ar-H), 7.42–7.38 (m, 4H, Ar-H); ^13^C NMR (100 MHz, chloroform-*d*_6_) δ (ppm) = 138.5, 133.8, 129.3, 128.3; LCMS (*m*/*z*): 224 (M + H); Formula: C_12_H_8_Cl_2_.

**6m [27]**: ^1^H NMR (400 MHz, chloroform-*d*_6_): δ (ppm) = 7.38 (d, *J* = 7.8 Hz, 4H, Ar-H), 7.31 (t, *J* = 7.1 Hz, 2H, Ar-H), 7.14 (d, *J* = 7.3 Hz, 2H, Ar-H); ^13^C NMR (100 MHz, chloroform-*d*_6_) δ (ppm) = 141.4, 138.3, 128.7, 128.0, 127.9, 124.2, 21.4; LCMS (*m*/*z*): 183 (M + H); Formula: C_14_H_14_.

**6q [27,66]**: ^1^H NMR (400 MHz, chloroform-*d*_6_): δ (ppm) = 9.83 (s, 2H, -CHO), 8.05 (d, *J* = 7.4 Hz, 2H, Ar-H), 7.66 (t, *J* = 7.1 Hz, 2H, Ar-H), 7.59 (t, *J* = 7.2 Hz, 2H, Ar-H), 7.36 (d, *J* = 7.4 Hz, 2H, Ar-H); ^13^C NMR (100 MHz, chloroform-*d*_6_) δ (ppm) = 191.2, 141.3, 134.7, 133.5, 131.8, 128.9, 128,7; LCMS (*m*/*z*): 211 (M + H); Formula: C_14_H_10_O_2_.

**6u [27,66]**: ^1^H NMR (400 MHz, chloroform-*d*_6_): δ (ppm) = 9.90 (s, 2H, -CHO), 7.71 (d, *J* = 3.8 Hz, 2H, HetAr-H), 7.41 (t, *J* = 3.8 Hz, 2H, HetAr-H); ^13^C NMR (100 MHz, chloroform-*d*_6_) δ (ppm) = 182.6, 144.8, 144.0, 136.9, 126.4; LCMS (*m*/*z*): 223 (M + H); Formula: C_10_H_6_O_2_S_2_.

## 4. Conclusions

To summarize, we developed a new, nature-inspired procedure for the aerobic self-coupling of PATFBs in water using the nitrogen-bridged polycyclic *N*-pincer Pd(II)-porphyrin complex, **PdTST*p*SPP,** as a safe catalyst at rt in open-air. This protocol showed advantages with the use of water as solvent, high TON and TOF values, low catalyst loading, large substrate feasibility, and avoidance of oxidant, base, phosphine ligands, and toxic solvents. To the best of our knowledge this is the first report on the use of metalloporphyrins for self-couplings of arylboron compounds.

## Figures and Tables

**Table 1:** Search for the catalyst and nucleophile ^1^.

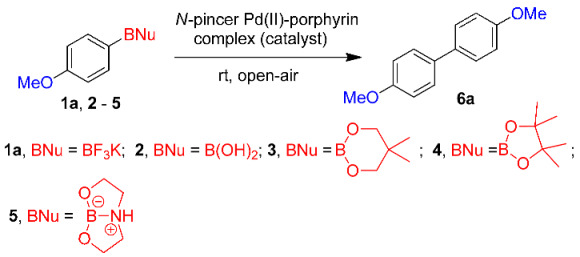					
Entry	Arylboron Compound	Catalyst (mol%)	Solvent (mL)	Time (min)	Isolated Yield (%)
1	**1a**	**PdTST*p*SPP** (0.1)	Water (4)	15	98
2	**1a**	**PdTPP** (0.1)	Water (2) + DMF (2)	240	21
3	**1a**	**PdT*p*AP** (0.1)	Water (2) + DMF (2)	240	39
4	**1a**	**PdT*m*HPP** (0.1)	Water (2) + DMF (2)	240	58
5	**1a**	**PdT*m*CPP** (0.1)	Water (2) + DMF (2)	240	67
6	**1a**	**PdTST*p*SPP** (0.07)	Water	15	98
7	**1a**	**PdTST*p*SPP** (0.05)	Water	15	98
8	**1a**	**PdTST*p*SPP** (0.03)	Water	60	82
9	**2**	**PdTST*p*SPP** (0.05)	Water	15	92
10	**3**	**PdTST*p*SPP** (0.05)	Water (2) + DMF (2)	180	14
11	**4**	**PdTST*p*SPP** (0.05)	Water (2) + DMF (2)	180	17
12	**5**	**PdTST*p*SPP** (0.05)	Water	60	73

^1^ Arylboron substrate (1 mmol) and 4 mL of solvent were used and the reactions conducted in open-air at rt.

**Table 2:** Substrate feasibility study ^1^.

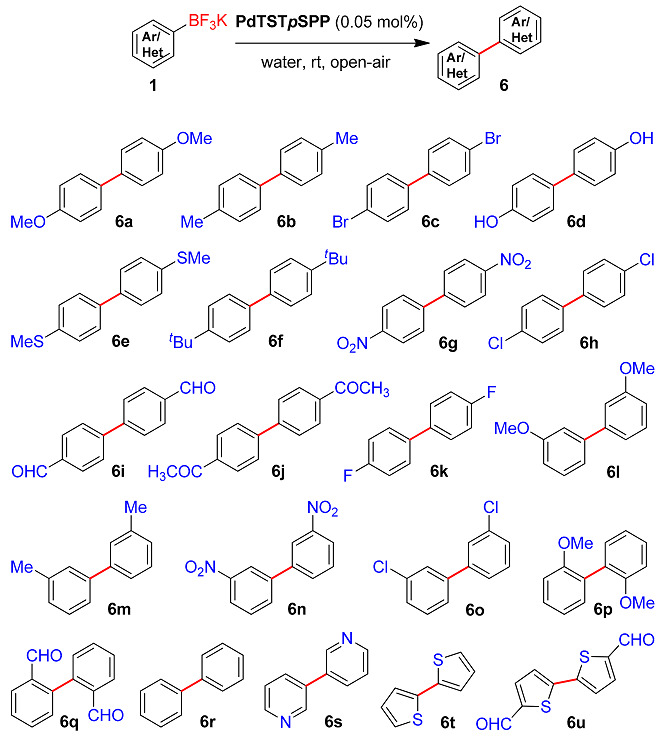						
Entry	Aryltrifluoroborate (1)	Time (min)	Product (6)	Yield (%) ^2^	TON	TOF
1	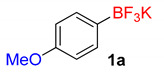	15	**6a**	98	1960	7840
2	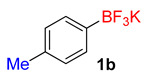	25	**6b**	94	1880	4512
3	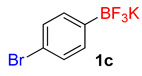	25	**6c**	92	1840	4416
4	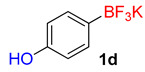	35	**6d**	89	1780	3051
5	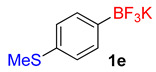	20	**6e**	94	1880	5640
6	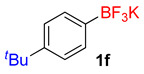	20	**6f**	94	1880	5640
7	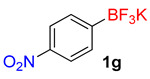	10	**6g**	99	1980	11,880
8	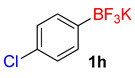	15	**6h**	97	1940	7760
9	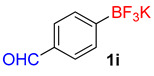	10	**6i**	99	1980	11,880
10	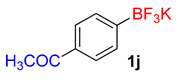	20	**6j**	97	1940	5820
11	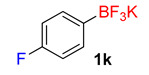	10	**6k**	98	1960	11,760
12	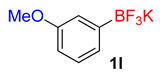	30	**6l**	93	1860	3720
13	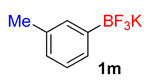	30	**6m**	88	1760	3520
14	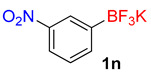	20	**6n**	95	1900	5700
15	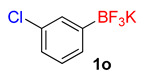	30	**6o**	92	1840	3680
16	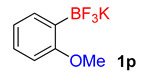	45	**6p**	88	1760	2347
17	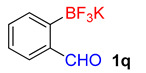	40	**6q**	91	1820	2730
18	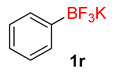	15	**6r**	96	1920	7680
19	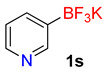	60	**6s**	91	1820	1820
20	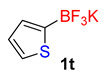	60	**6t**	86	1720	1720
21	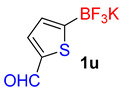	50	**6u**	90	1800	2160

^1^ Conditions: PATFB (1 mmol), water (4 mL) at ambient conditions in open-air. ^2^ Isolated yield.

**Table 3:** Comparison of self-couplings of aryltrifluoroborates.

Entry	Catalyst	Solvent	Base/Additive	Temp.	Time (h)	Yield (%)	Ref.
1	Pd NPs/Te-Dps ^1^	Water	Tris-HCl buffer	100 °C	10–24	60–87	[39]
2	Au nanoclusters:poly(*N*-vinyl-2-pyrrolidine)	Water	pH 6.86 buffer	47 °C	24	14–quant.	[40]
3	Pd(OAc)_2_–electrolysis	DMF	*p*-Benzoquinone	80 °C	0.24–0.40	41–99	[41]
4	Cu(OAc)_2_–ultra sound	Aq. EtOH	Dowex polymer support	Ultrasound	6	0–98	[42]
5	Pd NPs@Al(OH)_3_	Water	KOAc, Ag_2_O	50 °C	16–48	42–98	[43]
6	**PdTST*p*SPP**	Water	-	rt	0.17–1.0	86–99	Present

^1^ Pd NPs/Te-Dps; Pd nanoparticles stabilized with Dps protein of *Thermosynechoccus elongatus* bacterium.

## Data Availability

Not applicable.

## Supplementary Materials

The copies of ^1^H and ^13^C-NMR spectra of symmetrical biaryls are available online.

## Sample Availability

Samples of the compounds **6a**–**u** and **7** are available from with authors.
