# Supplementary material for: Porphyrin N-Pincer Pd(II)-Complexes in Water: A Base-Free and Nature-Inspired Protocol for the Oxidative Self-Coupling of Potassium Aryltrifluoroborates in Open-Air
Source: Molecules. 2021 Sep 4;26(17):5390. doi: 10.3390/molecules26175390 (PMC8433652; doi:10.3390/molecules26175390)

Porphyrin *N*-Pincer Pd(II)-Complexes in Water: A Base-Free and Nature-Inspired Protocol for the Oxidative Self-Coupling of Potassium Aryltrifluoroborates in Open-Air

Sana Siva Prasad[1], Bandameeda Ramesh Naidu[1], Marlia M. Hanafiah[2,3], Jangam Lakshmidevi[1], Ravi Kumar Marella[4], Sivarama Krishna Lakkaboyana[5] and Katta Venkateswarlu[1,*]

1 Laboratory for Synthetic & Natural Products Chemistry, Department of Chemistry, Yogi Vemana University, Kadapa 516005, India; sana.chemist009@gmail.com (S.S.P.); brameshnaidu94@gmail.com (B.R.N.); devi703244@gmail.com (J.L.)
2 Department of Chemistry, Faculty of Science and Technology, Universiti Kebangsaan Malaysia, Bangi 43600, Selangor, Malaysia; mhmarlia@ukm.edu.my
3 Centre for Tropical Climate Change System, Institute of Climate Change, Universiti Kebangsaan Malaysia, Bangi 43600, Selangor, Malaysia
4 Department of Mechanical Engineering, PACE Institute of Technology & Sciences, Ongole 523272, India; ravikumar.marella@pace.ac.in
5 Department of Chemical Technology, Chulalongkorn University, Pathumwam, Bangkok, Thailand; svurams@gmail.com
* Correspondence: kvenkat@yogivemanauniversity.ac.in; Tel.: +91-7989951388

Copies of ^{1}H & ^{13}C NMR Spectra of symmetrical biaryls:

^{1}H NMR spectrum of **6a**:

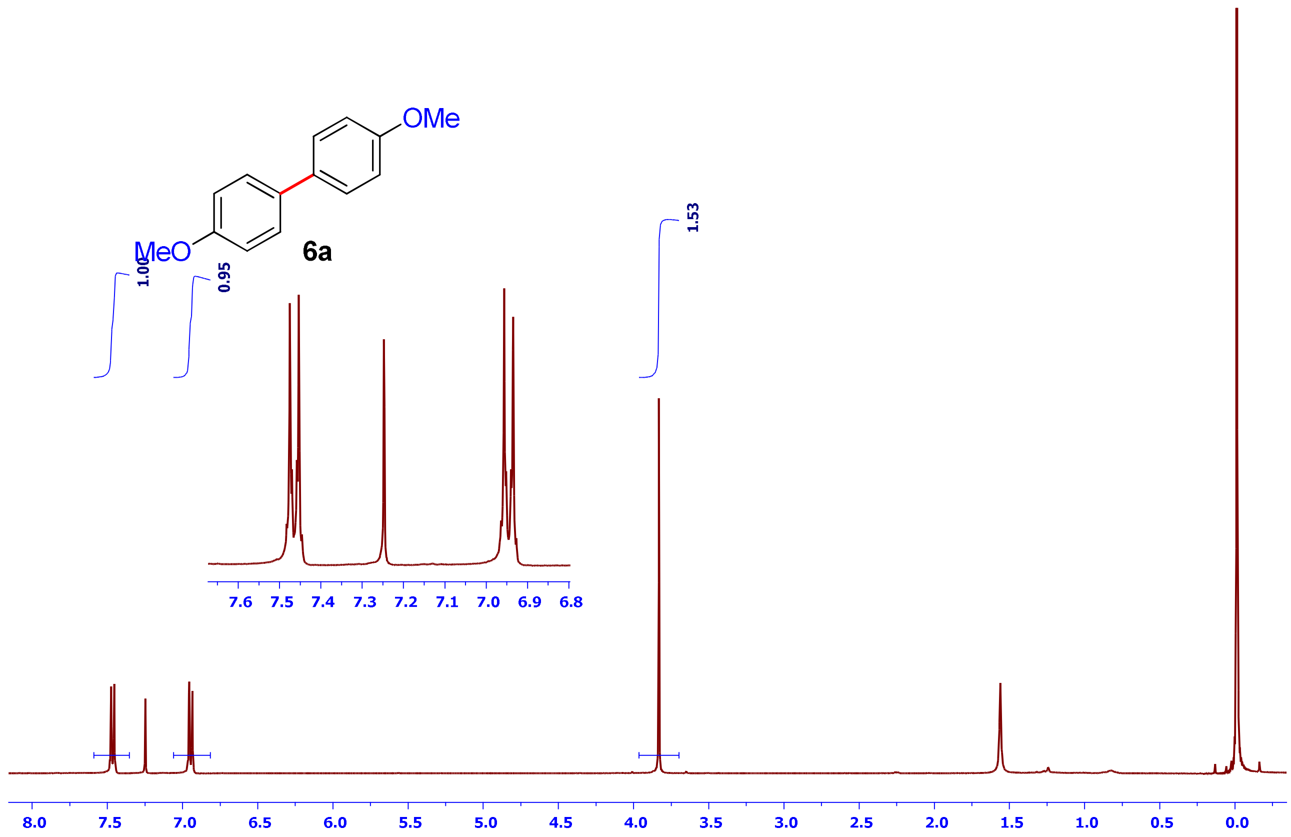

^{13}C NMR spectrum of **6a**:

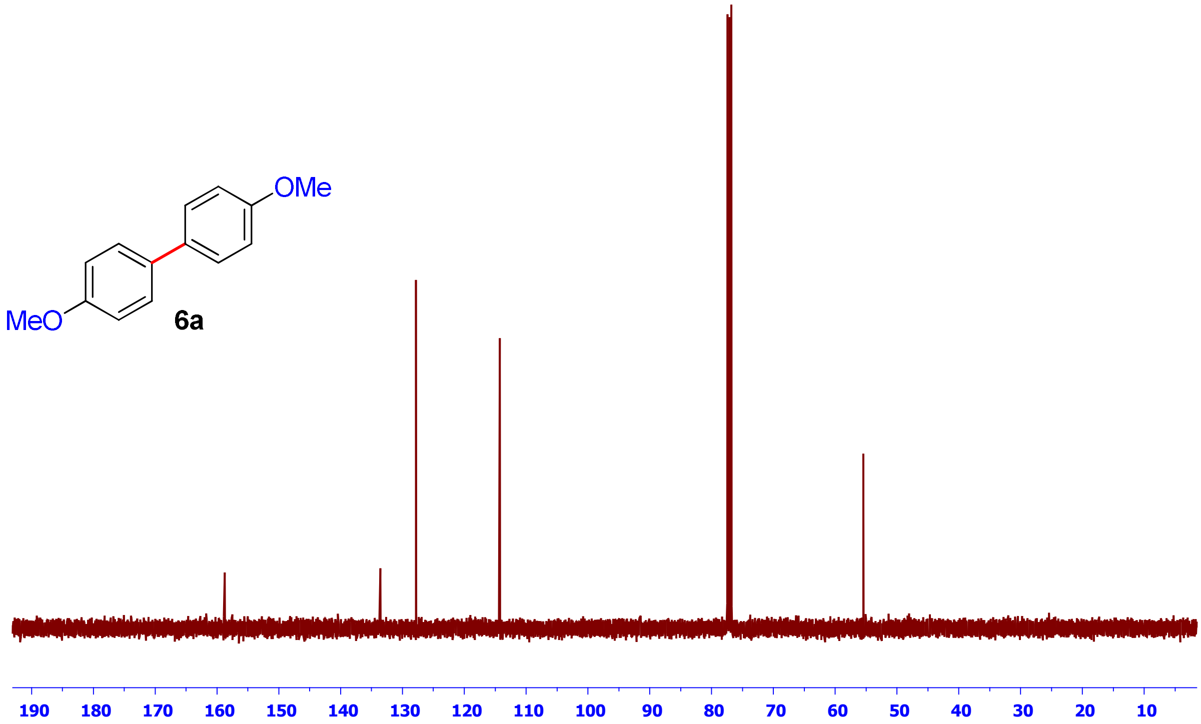

^{1}H NMR spectrum of **6e**:

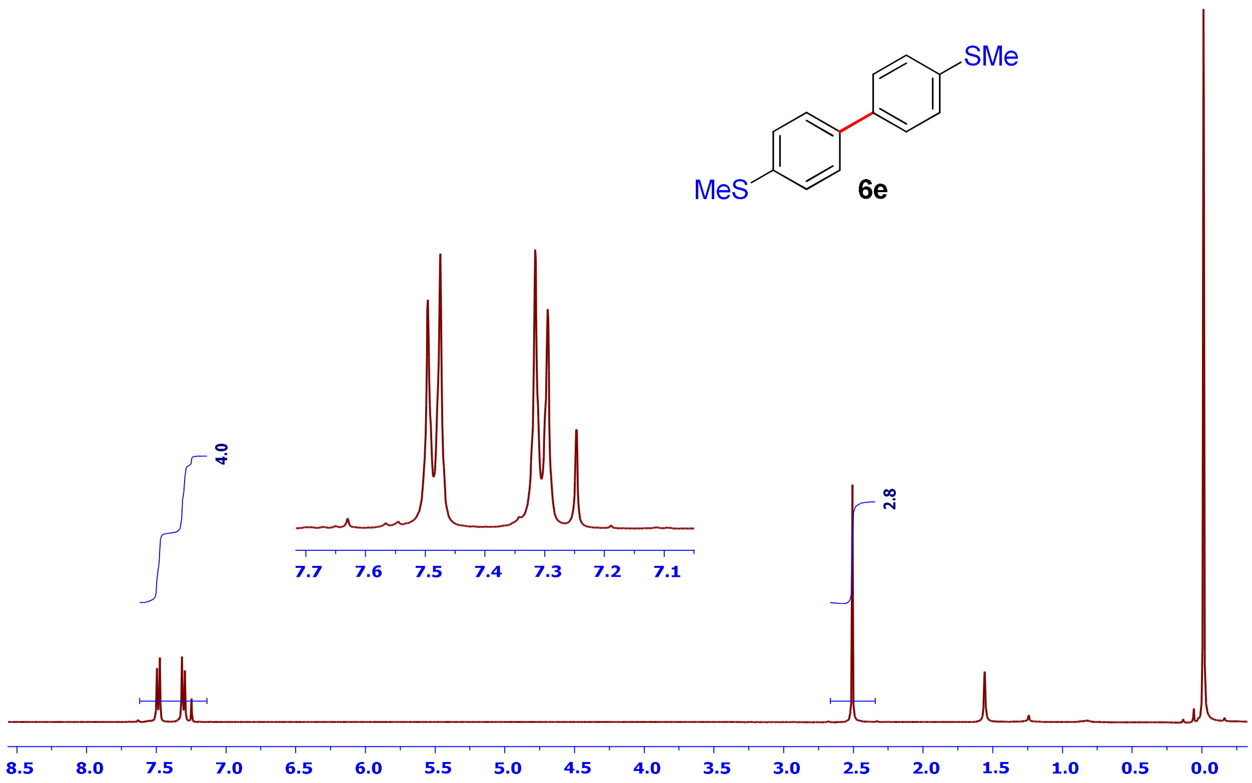

^{13}C NMR spectrum of **6e**:

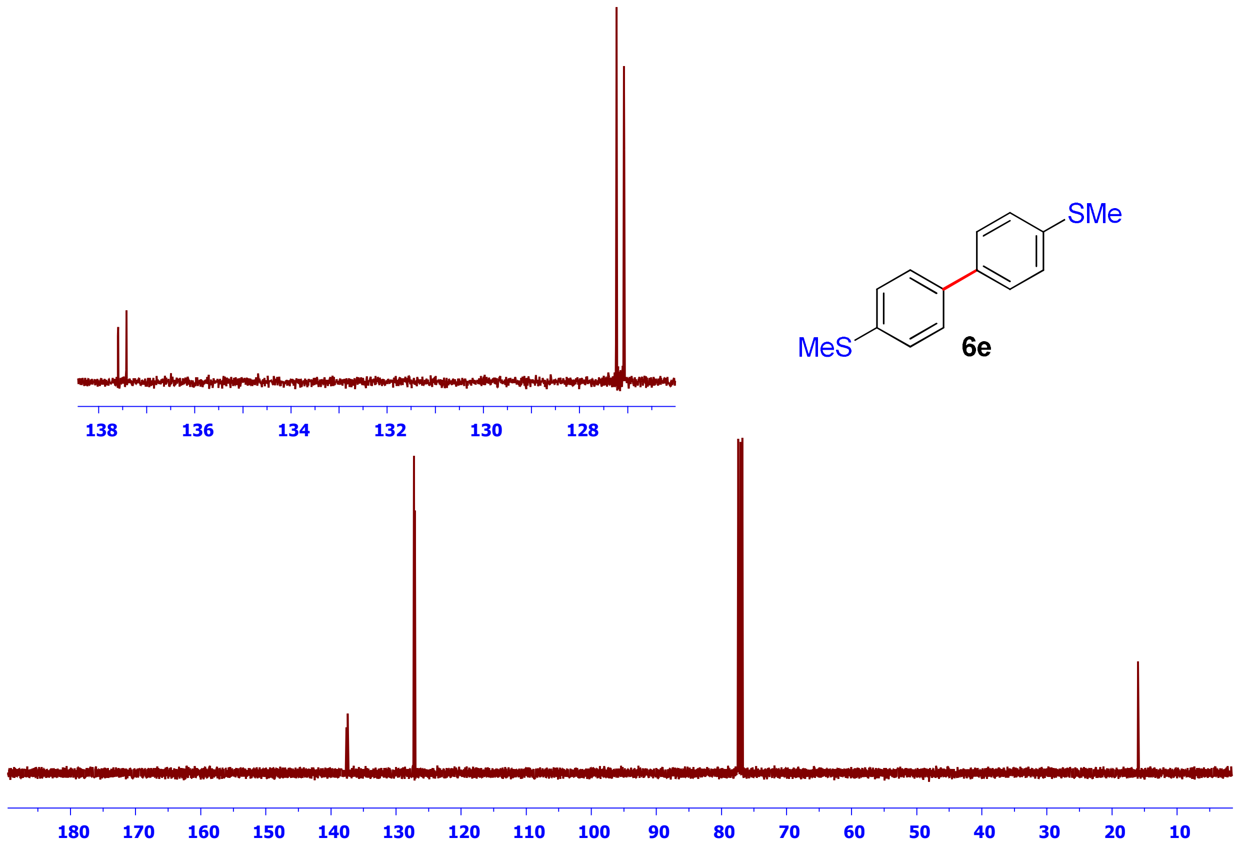

^{1}H NMR spectrum of **6f**:

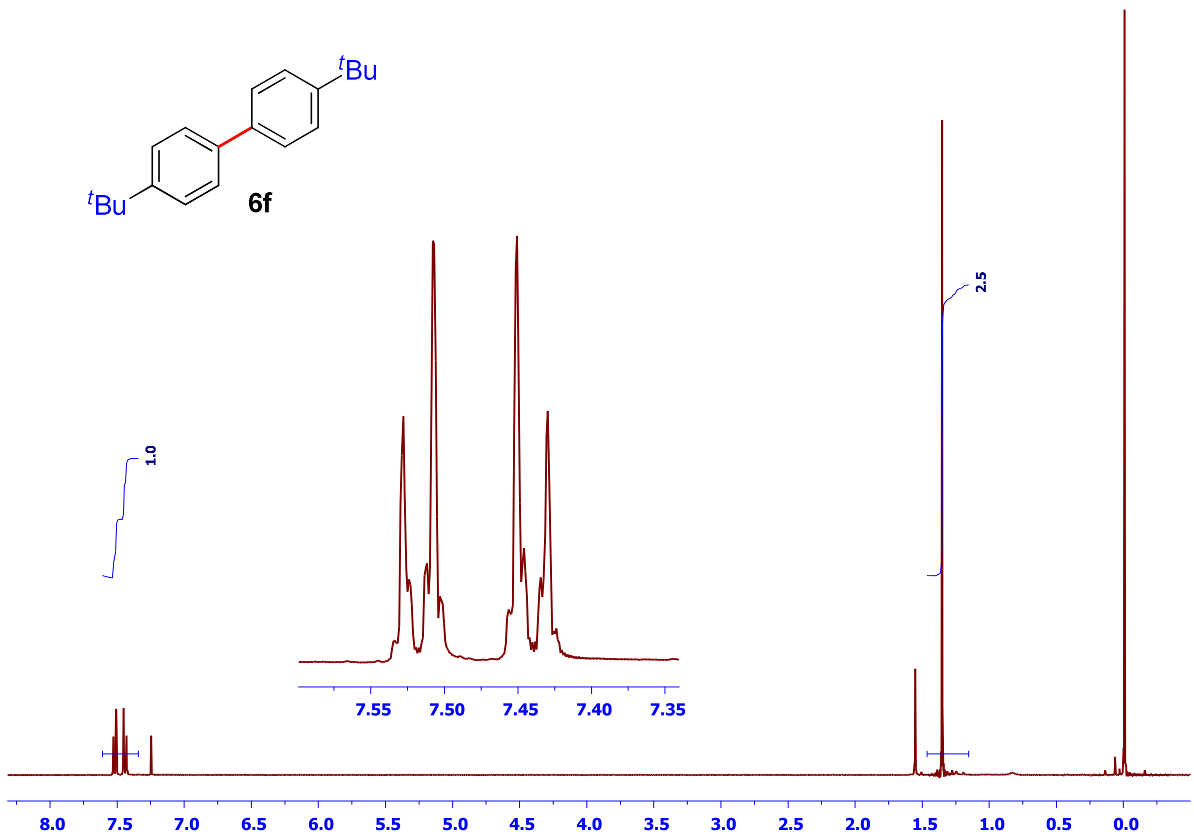

^{13}C NMR spectrum of **6f**:

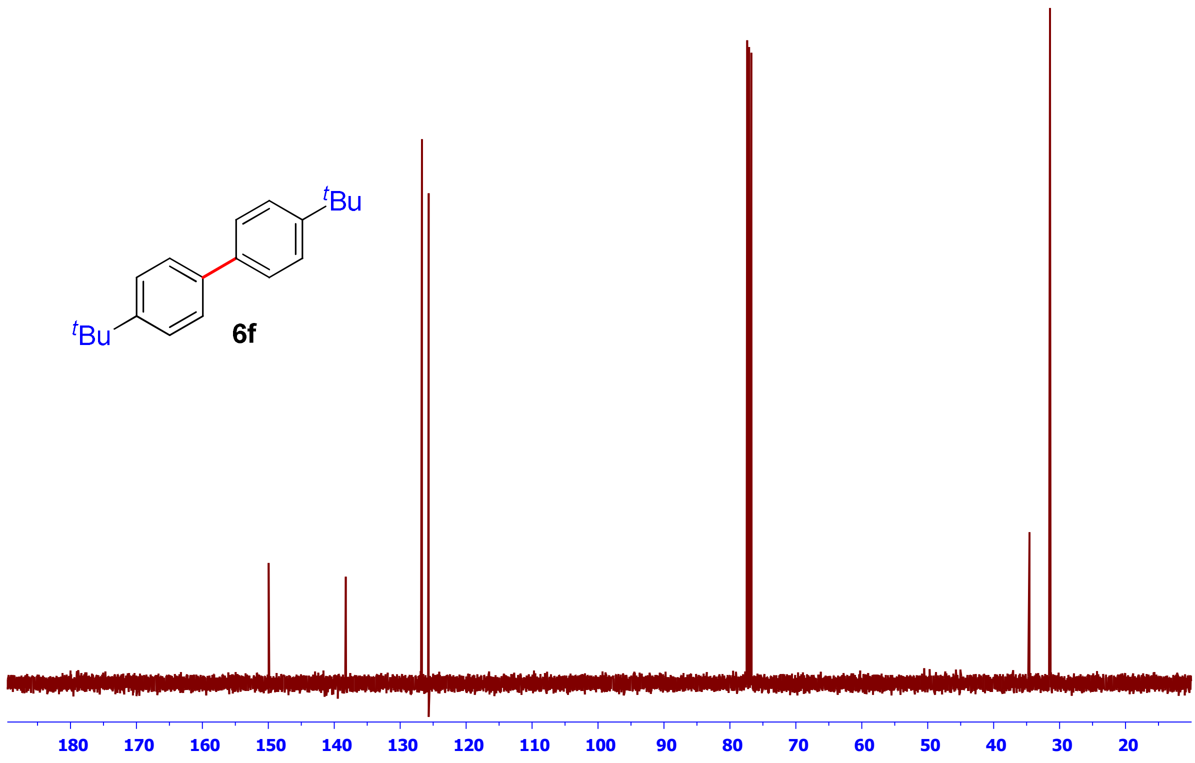

^{1}H NMR spectrum of **6h**:

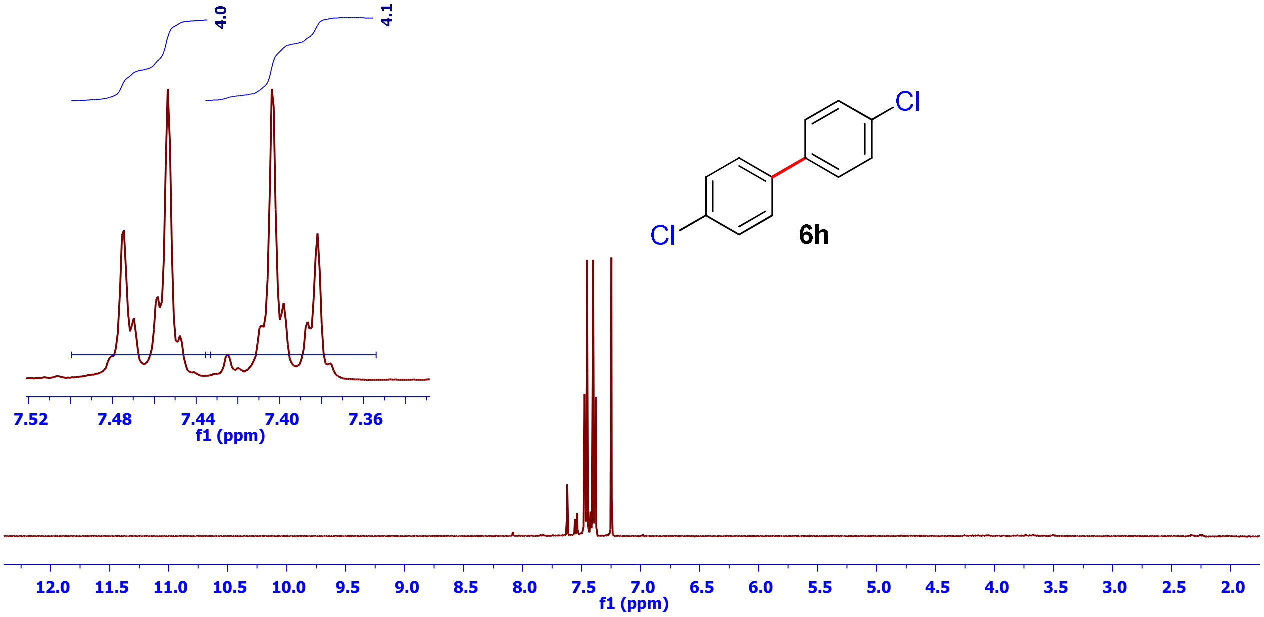

^{13}C NMR spectrum of **6h**:

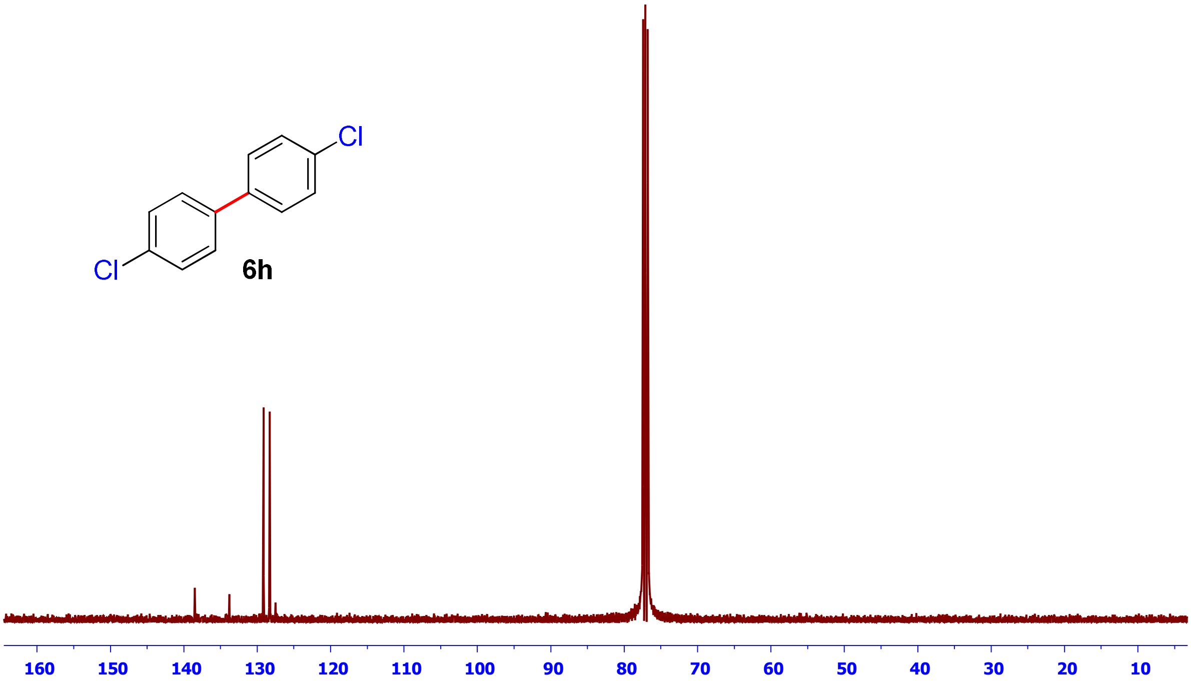

^{1}H NMR spectrum of **6m**:

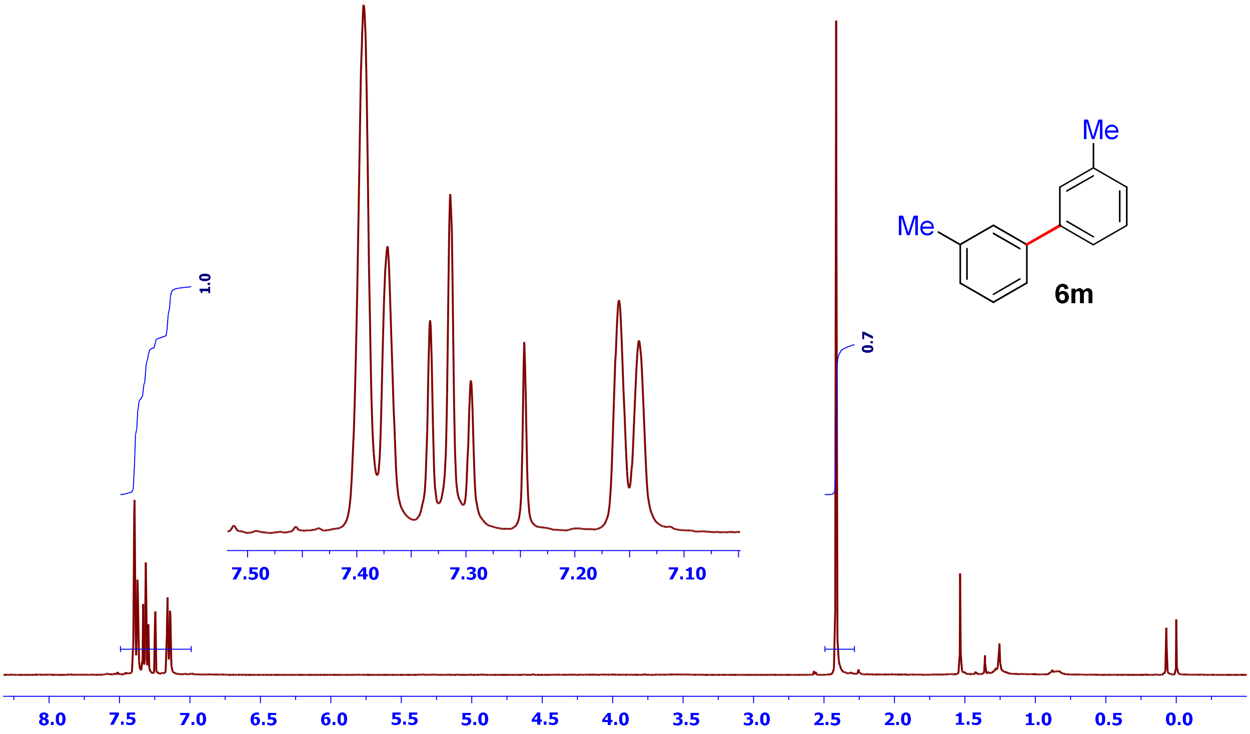

^{13}C NMR spectrum of **6m**:

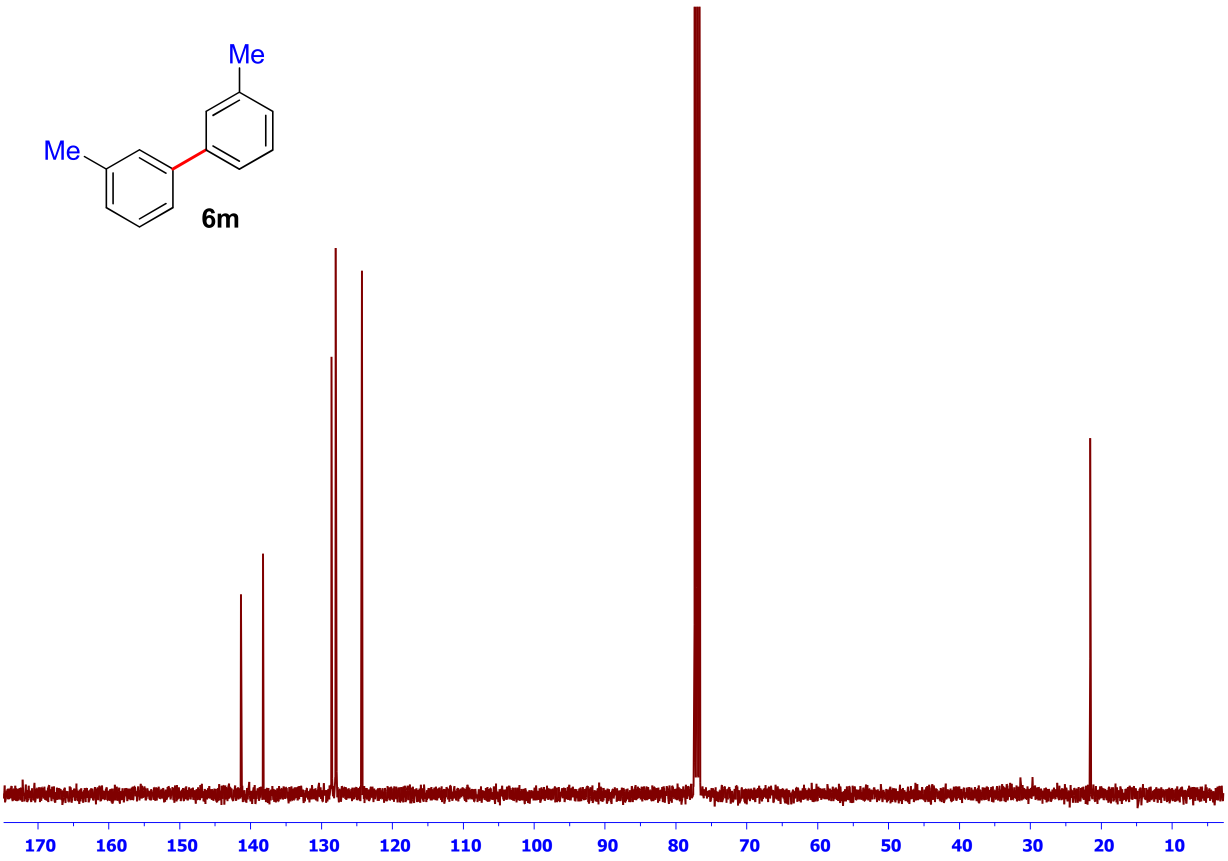

^1H NMR spectrum of **6q**:

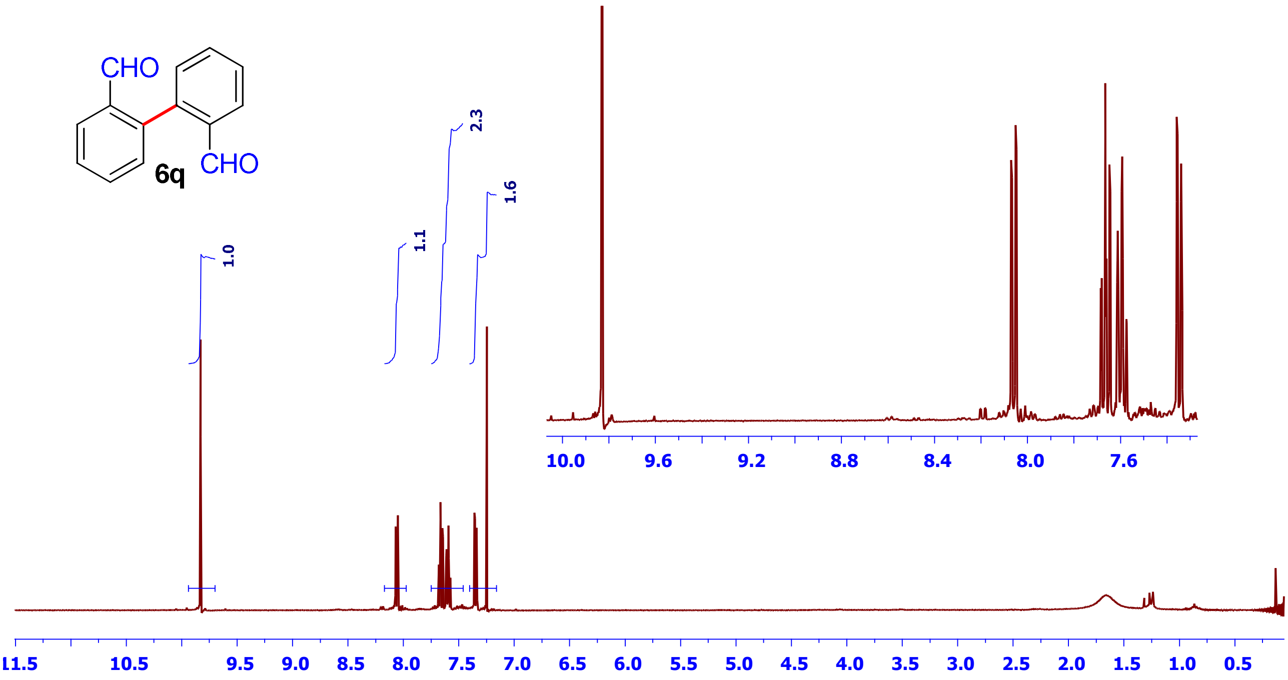

^{13}C NMR spectrum of **6q**:

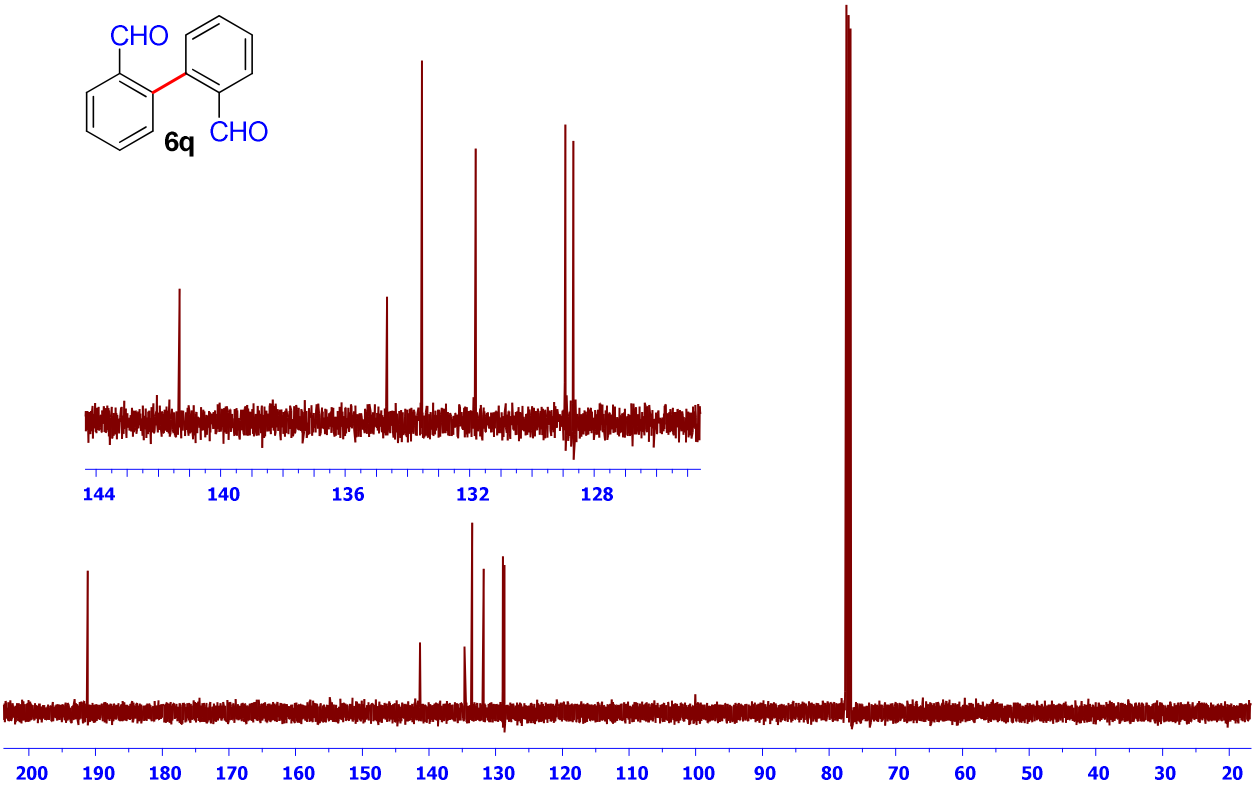

^{1}H NMR spectrum of **6u**:

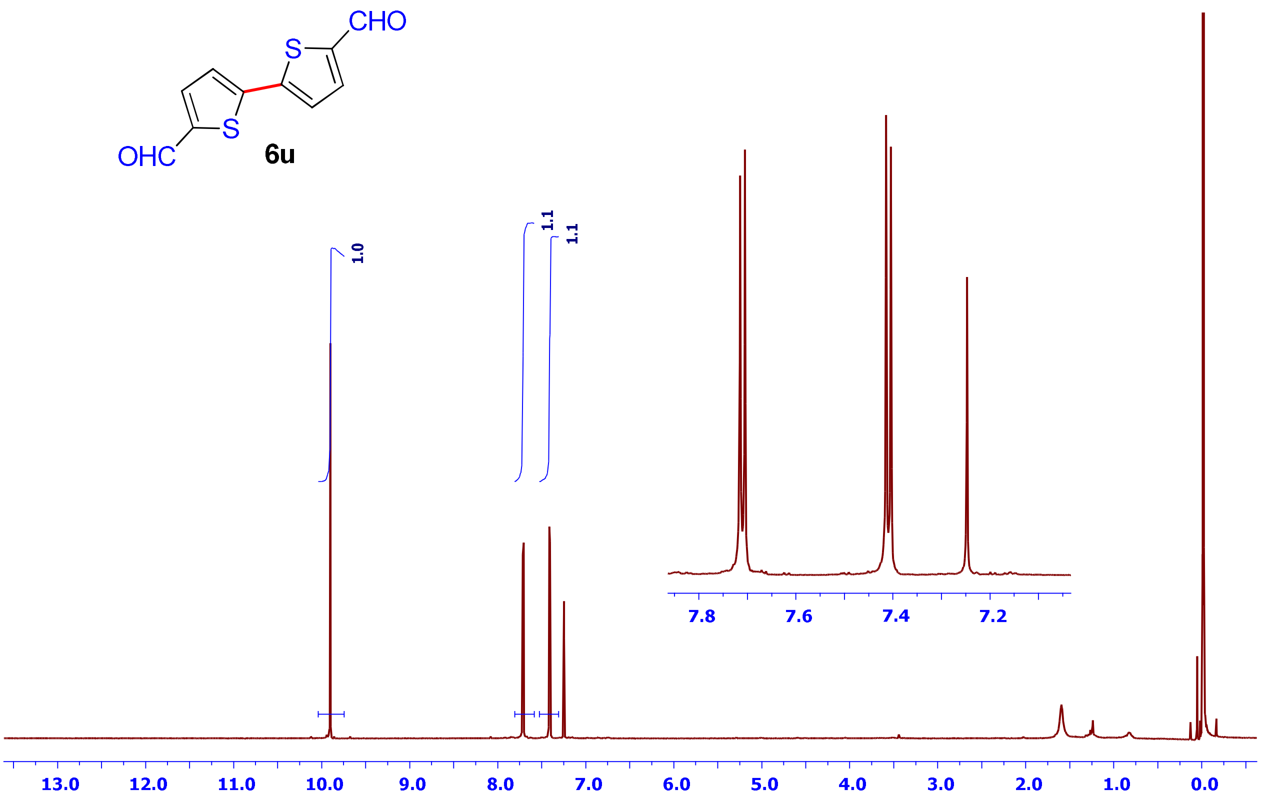

^{13}C NMR spectrum of **6u**:

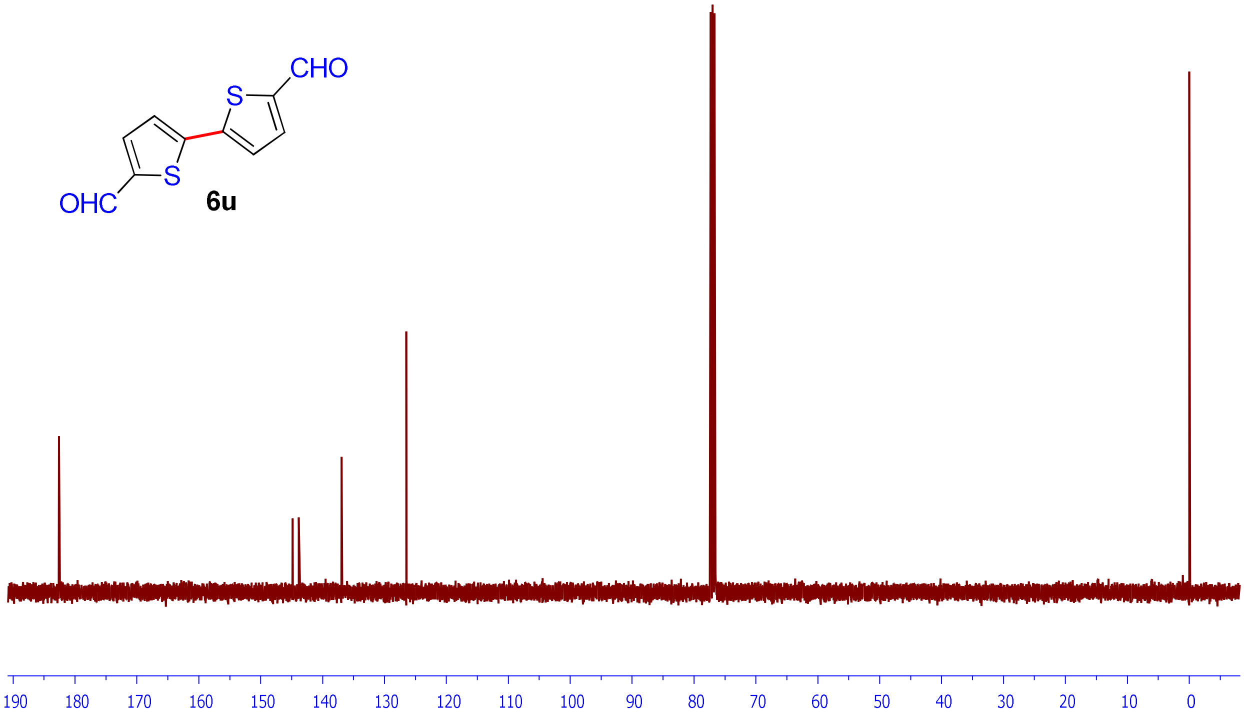

Supplement: Supplementary file 1 [file molecules-26-05390-s001.zip › molecules-1343647-supplementary.pdf]
